# The Role of Barriers and Community-Based Education in Compliance to Regular Eye Exams in New York City’s Harlem Community

**DOI:** 10.7759/cureus.10875

**Published:** 2020-10-10

**Authors:** Khaled Moumneh, Jenifer Centeno Gavica, Mishelle S Centeno Gavica, Mark Terrell, Christine M Lomiguen

**Affiliations:** 1 Primary Care, Touro College of Osteopathic Medicine, New York, USA; 2 Medical Education, Lake Erie College of Osteopathic Medicine, Erie, USA; 3 Pathology, Lake Erie College of Osteopathic Medicine, Erie, USA

**Keywords:** vision screening, health fair, visual health, community outreach, education, ophthalmology

## Abstract

Background

The American Optometric Association (AOA) recommends adults between the ages of 18 to 65 have an eye exam every two years while older individuals or those who utilize vision correction should have yearly exams. Previous vision screenings throughout New York City’s Harlem community determined that 60% warranted referral to an ophthalmologist.

Objective

As delayed vision screening could potentially leave a sight-threatening condition undiagnosed for many years, the objective of this study was to identify barriers to regular eye examinations.

Methods

A voluntary anonymous survey was verbally administered and recorded at free medical student-run vision screenings throughout multiple Harlem community health fairs. Data gathered included demographics, insurance coverage, comfort with an eye exam, and knowledge associated with an ophthalmologist eye exam. As a strategy to curtail the frequency of non-compliance to regular eye examinations, all participants were then educated about knowledge of ocular examinations and assured about uncertainties. Comfort levels were remeasured after education to determine the effectiveness and impact of community-based education.

Results

One hundred surveys fit the inclusion criteria. Of the participants with suboptimal knowledge of an eye exam (n=41), 32% were more comfortable after education and assurance versus 3% of the optimal knowledge participants (n=59). Of the participants with non-compliance to regular eye exams (n=39), 41% had suboptimal knowledge and 23% were more comfortable after education versus 33% and 10% of the compliant participants (n=61), respectively. Participants with suboptimal knowledge were 20.9% more likely to be noncompliant with regular eye examinations and at the same time, 18.1% more likely to have increased comfort after education and assurance than those with optimal knowledge. Of the participants without medical insurance (n=15), 67% were noncompliant with regular eye examinations and 60% had suboptimal knowledge versus 34% and 38% of the insured participants (n=85), respectively. Participants with medical insurance were 23.8% more likely to be compliant with regular eye examinations than those without medical insurance.

Conclusion

Measured perceptions of ophthalmology in New York City’s Harlem community solidifies a lack of education as a clear barrier against proper vision care. Lack of health insurance coverage also contributed to decreased participation in regular eye examinations but to a lesser extent than education. These results suggest that empowering people through education can significantly improve compliance with regularly scheduled eye exams, thus improving the overall visual health of this minority-rich community.

## Introduction

Vision problems are widespread and common in the United States of America (USA). According to the Centers for Disease Control and Prevention (CDC), as of 2012, 4.2 million Americans aged 40 years and older suffer from uncorrectable vision impairment. The prevalence is predicted to more than double by 2050 due to increased diabetes, hypertension, rapid aging of the population, and other chronic diseases that lead to vision loss through a variety of mechanisms [1]. Vision screenings, conducted either through community health fairs or primary care providers, are an important tool allowing earlier vision-saving intervention [2-4]. Current guidelines set by the American Optometric Association (AOA) recommend healthy adults 18-64 undergo eye exams at least every two years, while vision impairment, disease, risk factors such as diabetes, and those 65 and over require at least annual visits [5]. Based on student-run health fair data from previous vision screenings within New York City’s Harlem community, 60% warranted referral to an ophthalmologist [4].

As improved ocular health is desired, it is important to understand some of the barriers to accessing appropriate vision care especially in underserved minority communities such as those found in the Harlem neighborhood of Manhattan [6]. Frequently cited barriers for eye care include the cost of treatment, trust, communication, accessibility, and doctor-patient relationships [7]. Understanding barriers and developing strategies to mitigate the barriers can provide support for further improvement to community vision health [2,3].

The purpose of this study was to determine the extent to which lack of knowledge of eye examinations and health insurance coverage are barriers for eye screening and whether education about eye examinations can improve participant comfort levels for eye examinations within the Harlem community.

## Materials and methods

Free vision screenings were held at five health fairs at different sites throughout the Harlem neighborhood of New York, NY in the Fall of 2018. The health fairs utilized were at the Murid Mosque Health Fair, Health First Health Expo, New York Ephesus Seventh Day Adventist Church, Touro College of Osteopathic Medicine Fall into Health Fair, Williams Institutional C.M.E. Church, and Harlem Day Health Fair. Attendees were given the option to participate in a survey prior to vision screening. Congruent with Touro College’s Health Sciences Institutional Review Board for the Protection of Human Subjects (HSIRB #1784E), participants were not incentivized to participate in the survey with recruitment being completely voluntary. Vision screenings did not require participation in surveys, and participants had to be over the age of 18 and able to functionally communicate. 

A 10-question mixed multiple-choice and short-answer survey was developed specifically for this study to measure participant knowledge, comfort, and compliance with regular eye exams (Table 1). Surveys were administered by first and second year osteopathic medical students from Touro College of Osteopathic Medicine in conjunction with a Touro student organization, Otolaryngology and Ophthalmology Touro Interest Club (OOPTIC). All students were trained by the research co-investigators in a 30-minute training session and deemed qualified to administer the survey orally. Survey questions were available in both English and Spanish and were given orally by administrators fluent in the respective languages with anonymity assured. Participants were not shown the survey and instead were posed the questions in the form of a structured interview conversation. Demographic information such as gender, age, medical insurance, ethnic or racial group, and current vision health status were collected. Participant date of last eye exam was noted followed by a reason if more than a year or two had passed, depending on their recommended vision screening frequency [5]. Comfort level associated with receiving an eye exam by an ophthalmologist was assessed on a scale of 1-10, with 10 denoting “very comfortable”, followed by the assessment of their general knowledge of an ophthalmology visit. Education was then offered either by filling gaps in knowledge or other assurances with an original document based on the American Academy of Ophthalmology (AAO) online information on eye exams for patients (Table 2) [8]. Following education, another comfort level on a scale of 1-10 was obtained. Patient knowledge was qualified as optimal or suboptimal based on an original eight-point scale created from major categories of the AAO online information on eye exams for patients (Table 2). Compliance was based on AOA Exam guidelines [5]. Statistical analyses included chi-square, Mann-Whitney U-test, and correlation, which were all conducted using Statistical Package for the Social Sciences (SPSS) Statistics Software (IBM: Armonk, NY).

**Table 1:** Questionnaire outline The questionnaire was administered verbally in either English or Spanish as needed and filled out by an administrator

Questions
1. Sex (Male, Female)
2. Age
3. Medical insurance (Yes or No)
4. Ethnic/Racial Group (Black, Hispanic, White, Asian, Other)
5. Do you wear corrective vision equipment (glasses/contacts) or have other vision related problems (i.e. pain, glaucoma, diabetes)? (Yes or No)
6. When was your last eye exam?
7. If it has been more than a year, why?
8. What is your comfort level associated with going to an ophthalmologist for a regular check-up on a scale of 1-10, 10 being very comfortable?
9. Can you explain to me what happens at a regular eye exam?
10. After receiving more information on eye exams, what is your comfort level now on a scale of 1-10? 10 being very comfortable?

**Table 2:** Qualifying scale for knowledge A perfect score of 8/8 is considered optimal knowledge. A score of less than eight is suboptimal. Participants were hinted and prompted for expansion on missed points once before losing the point

Knowledge	Points
History or discussion of eye health	1
Eye chart or vision check	1
Peripheral eye exam	1
Mydriatic Drops	1
General eye exam (any mention of a handheld scope, light, or extraocular movement check was accepted)	1
Phoropter (recognition of the description of the device or use of different lenses was accepted).	1 (point given regardless of knowledge if participant did not require vision correction)
Tonometry (puff of air, touching of the eye with a device, or blue light was accepted)	1
Slit Lamp Exam (any mention of the device or examination was accepted)	1
Total	8

## Results

A total of 108 Harlem community members participated in the survey with eight exclusions due to non-completion or missing data, yielding a final sample size of 100. A summary of the demographic and descriptive data is presented in Table 3. Concerning demographics, two-thirds of the participants were female, and three-fourths of the participants were African American. Concerning health, 85% of the participants had medical insurance, 68% of the participants reported vision problems, and 61% indicated they were compliant with regular eye examinations. Concerning knowledge of eye examinations, although 41% were found to have optimal knowledge of eye examination processes and procedures, average participant knowledge was 5.59/8 as measured by our original scale. Concerning participant comfort level, participants rated their comfort level with eye examinations highly (9.01/10 average).

**Table 3:** Descriptive statistics (n=100)

Demographics and Patient Statistics	
Gender	Male: 35%, Female: 65%
Ethnicity	Black: 75%, Hispanic: 16%, White: 3%, Asian: 0%, Other 6%
Medical Insurance	Yes: 85%, No: 15%
Problems with vision (glasses or other issues)	Yes: 68%, No: 31%
Compliance with regular eye exams	Yes: 61%, No: 39%
Optimal knowledge	Yes: 59%, No: 41%
Average Participant Knowledge	5.59 (out of 8)
Positive change in comfort after education	Yes: 15%, No: 85%
Average comfort level before education	9.01 (out of 10)
Average comfort level after education	9.37 (out of 10)

Analyses involving identifying predictors of participants’ noncompliance with eye examinations included participants’ lack of knowledge of the examination process and lack of medical insurance (Table 4). Concerning knowledge of the eye examination, of the 61 participants who self-reported as being compliant with regular eye examinations, only 33% of these participants had suboptimal knowledge as measured by our original scale. In contrast, of the 39 participants who indicated noncompliance with regular eye examinations, 54% of these participants had suboptimal knowledge of eye examinations. Concerning medical insurance, 85 participants indicated they had medical insurance, but 34% were noncompliant with regular eye exams and 38% had suboptimal knowledge. Of the 15 participants who lacked health insurance coverage, 67% of these participants were noncompliant and 60% exhibited suboptimal knowledge.

**Table 4:** Summary of frequencies for different subsets of participants (n=100)

	Positive change in comfort	Suboptimal Knowledge	Noncompliant with regular eye exams
Suboptimal Knowledge (n=41)	13 (32%)	-	21 (51%)
Optimal Knowledge (n=59)	2 (3%)	-	18 (31%)
Noncompliant with regular eye exams (n=39)	9 (23%)	21 (54%)	-
Compliant with regular eye exams (n=61)	6 (10%)	20 (33%)	-
Medical Insurance (n=85)	11 (13%)	32 (38%)	29 (34%)
No Medical Insurance (n=15)	4 (27%)	9 (60%)	10 (67%)

As a strategy to increase future compliance with eye examinations, community-based education was provided to all study participants. Of those 41 participants identified through our original eye exam knowledge scale as having suboptimal knowledge of the eye examination, 32% had a positive change in comfort after education. However, and as expected, only 3% of participants having optimal knowledge of eye examinations reported an increase in comfort after education. Concerning the 39 participants who self-reported as being noncompliant with regular eye examinations, 23% of these participants had a positive change in comfort after education. Concerning the 61 participants who self-reported as being compliant with regular eye examinations, only 10% of these participants had a positive change in comfort in response to education (Table 4).

Statistical analysis of the significance of the predictors for compliance with eye examinations is detailed in Table 5. Participants with suboptimal knowledge regarding eye exams were found to be 20.9% less likely to be compliant with eye exams (p=.037). However, after community-based education on eye examinations, 39% of the participants who were originally identified as having suboptimal knowledge, were more likely to become comfortable seeking out and complying with eye examinations (p<.001). Also, participants with medical insurance were found to be 23.8% (p=.017) more compliant with eye exams comparted to those without insurance. However, community-based education for optimal knowledge participants who were noncompliant with eye exams did not lead to a positive change in comfort after education (18.1%, p=.072). Time since last eye exam had no significant effect on knowledge or comfort level before education.

**Table 5:** Correlation results from statistical analyses *Indicates significance (n=100)

Relationship	Correlation
Knowledge versus Compliance	.209* (p=0.037)
Knowledge versus positive change in comfort	-.390* (p<0.01)
Insurance versus compliance	.238* (p=0.017)
Compliance versus positive change in comfort	-.181 (p=0.072)
Comfort before education versus time to last eye exam (years)	.008 (p=0.352)
Insurance versus comfort before	.014 (p=0.195)
Last eye exam in years versus knowledge	-.153 (p=0.138)

## Discussion

A combined effort has been made in recent years to improve vision health in the USA. CDC in collaboration with the National Opinion Research Center developed the nation’s first Vision and Eye Health Surveillance System (VEHSS) to help patients, health professionals, researchers and policymakers understand the scope of vision loss, eye disorders, and eye care services in the USA [9]. However, with healthcare disparities still ever-present in minority communities, often attributed to differing levels of health literacy and language proficiency, proper ocular health education may not always be conveyed [10,11].

Although accessibility is an established barrier to eye care, access to eye care professionals was not specifically addressed in this study [7]. Utilizing an internet-based search, 34 practices matching ophthalmologists and optometrists were found in the Harlem area which encompasses both East and West Harlem serving an estimated population of 402,549 residents [12-14]. Nonetheless, this potential barrier should be addressed in future studies of this population.

From sampling members of the Harlem community, the biggest predictor of non-compliance with regular eye examinations was lack of knowledge of the importance and process of the examination, followed by the possession of health insurance. Although participants who were less informed about eye exams exhibited less compliance with regular eye exam recommendations, they were much more likely to benefit from community-based education on reducing knowledge gaps, abating fears of ocular care, and emphasizing the importance of visual health. Therefore, community-based education on human health, including eye examinations, is effective.

## Conclusions

In this original research study, we highlight lack of education as a barrier to seeking regular eye exams in the minority community of Harlem. Although insurance was also shown to play a modest role in compliance, knowledge of the ophthalmologist visit played a larger role. In closing, increased community-outreach education by medical students is an effective method to improve comfort with vision health and, therefore, can potentially increase community vision health.
